# 

**Published:** 1887-02

**Authors:** 


RECEIPTS.

For 1886.—Drs. J. H. Reynolds, E. A. Anderson, F. M. Beaty, J. O. Whatley, John H. Henry, J. B. Gresham, J. F. Earnest, J. T. Cleveland.

For 1887.—Drs. J. M. Stansill, L. H. Cartledge, W. B. King, T. L. Gipson, T. F. Bivins, John Gerdine, J. W. Baker, T. L. Quillian, P. C. Tircuit ; J. E. Wright, to October ; E. Thomas, J. R. Jenkins, M. E. Rain, S. E. Barton, K. L. Grimes, J. J. Harmon, J. H. Henry, J. E. Kintz, J. T. Renouff ; E. A. Anderson, J. C. Smith, to July ; John E. Miller, L. W. Pitchford, A. R. Stephens, A. L. Elder, W. L. Green, D. M. Wheeler, W. H. Aycock, J. A. Ison, T. D. McDaniel, W. J. Westbrook, J. A. S. Bailey, R. L. Boynton, R. A. Justice, A. J. Jordan, J. W. Hammond.

